# A golden period for environmental soil chemistry

**DOI:** 10.1186/s12932-020-00068-6

**Published:** 2020-04-01

**Authors:** Donald L. Sparks

**Affiliations:** Delaware Environmental Institute, University of Delaware, Newark, DE 19716 USA

In many respects, the field of environmental soil chemistry has never been more important than today. Many of the critical environmental issues we face globally are linked to the changing climate, which is having profound impacts on the chemistry of soils. We have a poor understanding of how climate impacts not only chemical, but also physical, biological, and mineralogical properties and processes of soils. Figure 1 shows some of the major impacts of climate change on soils and water. Soils, globally, are under immense stress due to erosion, nutrient imbalances, salinization, desertification, pollution and acidification [1]. Our very best soils are being lost to development. In short, the fate of our soils and human security are inextricably linked [2]. The population of the world stands at 7.5 billion. It is expected to rise to 9–9.5 billion by 2050 and perhaps to 11 billion by 2100. Megacities are sprouting up in many areas, particularly in Asia. These are cities of more than 10 million people. Much of the population growth is occurring in urban areas, in particular coastal regions. For example, more than 50% of the U.S. population lives in coastal areas. The latter areas are very susceptible to increased flooding and sea level rise.

**Fig. 1:**
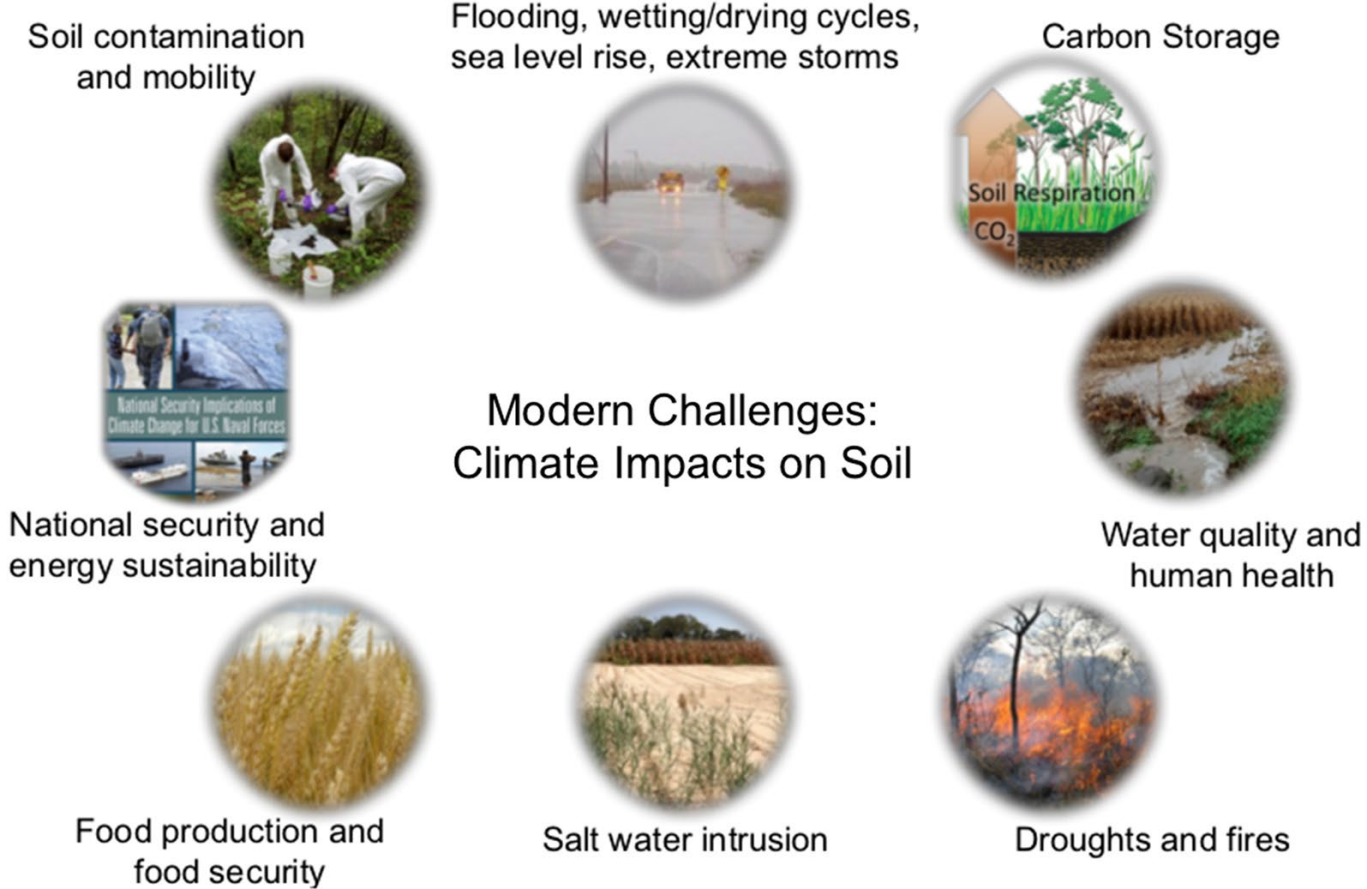
Climate change impacts on soils

With the impacts of climate and environmental change, there is incredible pressure to ensure an adequate food supply, especially for the most vulnerable regions, e.g., those in Africa. The production of enough food is dependent on adequate water, productive land, and in general healthy soils. A recent report from the Intergovernmental Panel on Climate Change [3] found that a half billion people live in locations that are seeing increased desertification and soils are being lost between 10 and 100 times faster than they are forming. Climate change will exacerbate these threats even more due to flooding, droughts, storms and other extreme weather events, further affecting the food supply. The report also notes that presently more than 10 percent of the world’s population is undernourished which could enhance cross-border migration and a quarter of humanity faces significant water crises.

Water quantity is particularly problematic with the increasing high temperatures and drought that we are seeing in areas such as the Western U.S., Africa, and many other parts of the world. Of the total water on Planet Earth, 96.5% is in oceans, bays, and glaciers. Groundwater, which is a major source of drinking water, comprises only 1.69% of the total water, and of this, only 0.76% is fresh water [4]. In a recent article in the New York Times [5], it was noted that 17 countries are under severe water stress. In addition to issues related to water scarcity, there are major challenges globally with water quality, related to excess nutrients such as nitrogen (N) and phosphorus (P) derived from organic wastes and inorganic fertilizers. In areas of high animal production, excess N and P in soils enter water bodies, causing hypoxia, resulting in algal blooms, fish kills and further impacts on tourism and even human health. Emerging organic contaminants such as antibiotics, hormones, per- and polyfluoroalkyl substances (PFAS), and others and their impact on drinking water, are also of great concern, particularly as populations increase. All of these contaminants impact human health and our economic vitality.

Carbon dioxide levels have been increasing at an alarming rate, particularly over the last few decades. Prior to the industrial revolution, CO_2_ levels were about 280 ppm. By 2019 they had risen above 410 ppm, levels that last occurred 3 million years ago. Human activities are estimated to have caused an approximately 1.0 ℃ rise in global warming above pre-industrial levels, with a probable range of 0.8–1.2 ℃, and are likely to reach 1.5 ℃ between 2030 and 2052 if global warming continues at the present rate [6] (Fig. 2). The last several years have been the warmest on record. Many scientists have called this geological period in history the Anthropocene as conclusive scientific evidence shows that humans are having a major impact on Planet Earth. As Aldo Leopold so insightfully noted in 1933, “The reaction of land to occupancy determines the nature and duration of human civilization”.

**Fig. 2:**
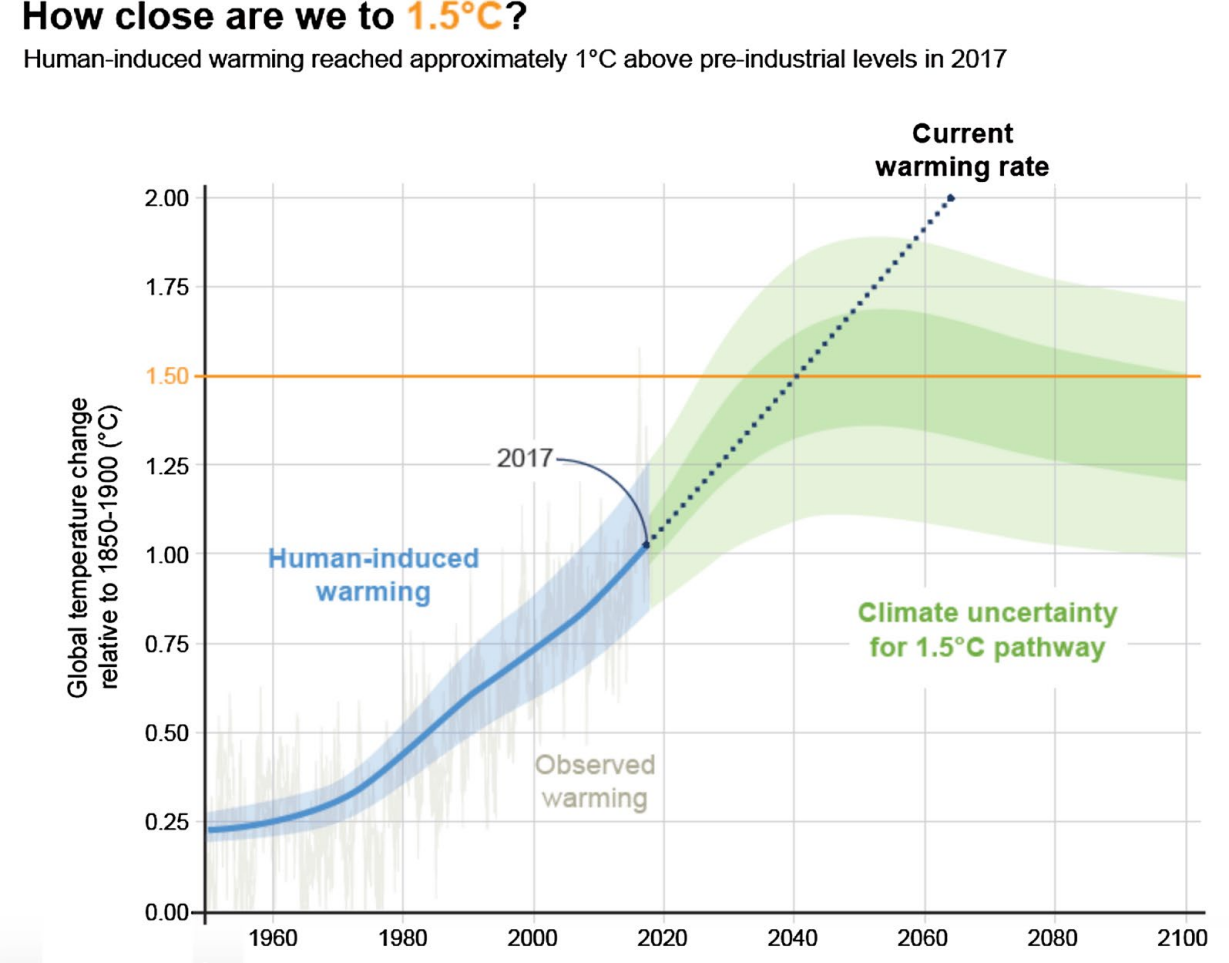
Global temperature change with time

The increases in greenhouse emissions and rising temperatures have resulted in melting glaciers, less snow cover, diminishing sea ice, rising sea levels, ocean acidification, and increasing atmospheric water vapor. Extreme events such as intense rainfall, heat waves, and forest fires, and droughts are becoming more frequent [7]. In terms of sea level rise, the global sea level has risen 0.18–0.20 m since 1900, with about half (0.08 m) of the rise occurring since 1993. The increasing sea level has resulted in more frequent flooding in coastal areas. Global average sea levels will continue to rise with model projections of a rise of 0.26–0.77 m by 2100 if global warming of 1.5 ℃ occurs [6]. The most vulnerable areas in the continental U.S. are along the Atlantic and Gulf Coasts. Subsidence, or land that is sinking, is compounding the problem, e.g., along the Mid-Atlantic Coast of the U.S. With increases in sea levels and flooding, there is increasing salinization of land and groundwater. Additionally, there are 2500 sites along the Atlantic and Gulf Coasts that are contaminated with metals, metalloids, and organic chemicals in areas that are heavily populated [8] (Fig. 3). It is not known how flooding and sea level rise, with its attendant salinity, will impact cycling of the contaminants and human health.

**Fig. 3:**
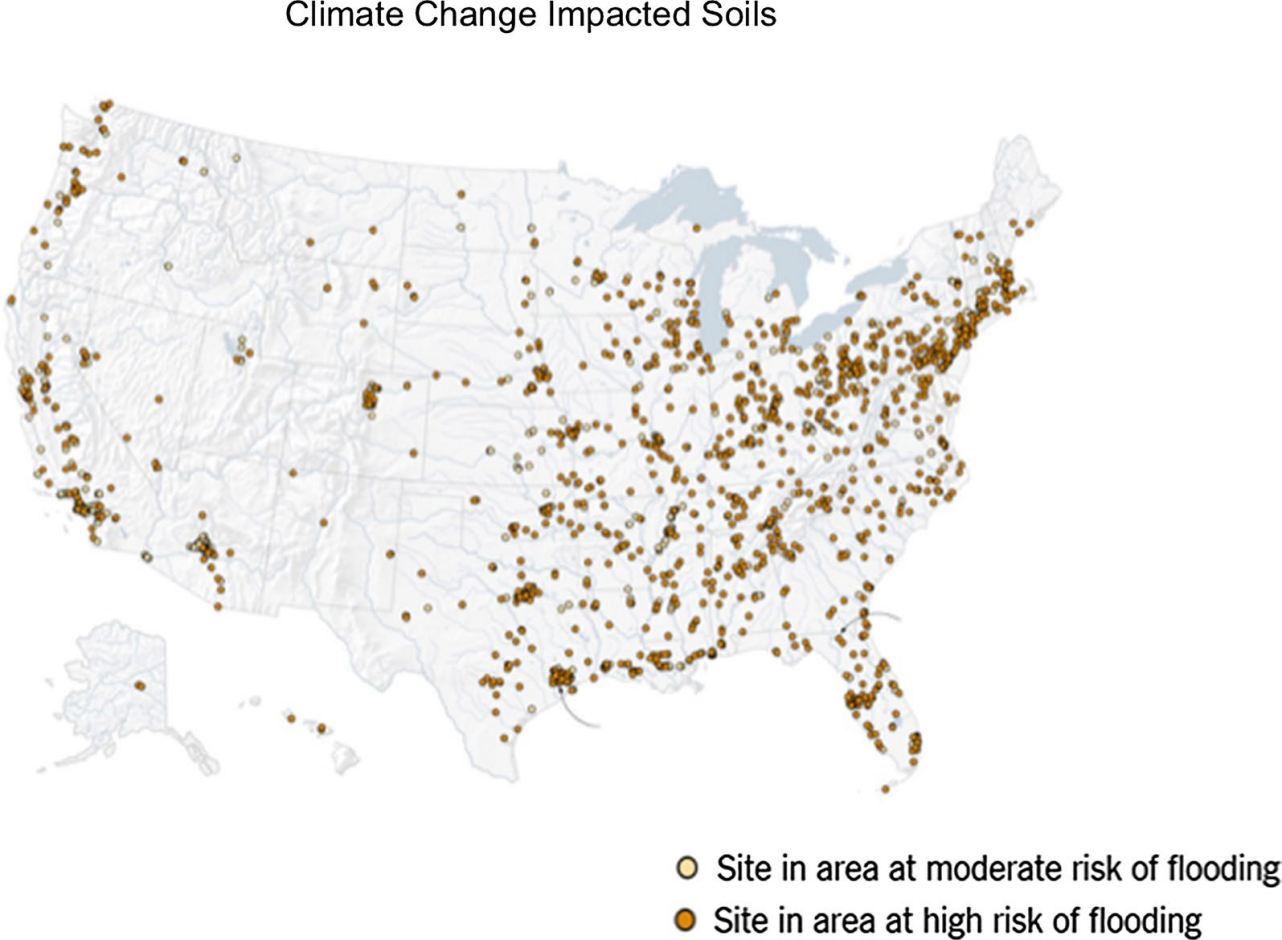
Contaminated sites in the U.S. which are subject to flooding

There is great concern about the impacts of rising temperatures on melting of permafrost soils. Permafrost soils sequester 1035 petagrams (Pg) of carbon (C) [9] in the top 3 m of soil, which represents about 70% of the current estimate for global soil C storage in the top 3 m (1500 Pg C) [10]. Research has already shown high labile C fractions released from permafrost soils that are thawing [11–13]. Plaza et al. [14], by quantifying C related to fixed ash content, measured soil C pool changes over a period of 5 years in warmed and ambient tundra ecosystems in Alaska. They found a 5.4% loss of C/year. They attributed much of the loss to lateral hydrological export. In a recent paper, Hemingway et al. [15] found that tightly mineral bound OC persists for millennia. It is critical to understand the role of warming in release of C, particularly C that is complexed with soil minerals such as iron oxides, which are major components for sequestering soil carbon [16–20].

## Major decadal research thrusts in environmental soil chemistry

In view of the above environmental challenges, it seems clear that the major research frontiers in environmental soil chemistry over the next 5–10 years will be heavily focused on the impacts of climate change on various soil chemical and mineralogical reactions and processes. Progress in these and other areas will result in large part due to rapid advances in analytical tools, data science, and modeling capabilities. As Nobel Laureate Sydney Brenner once said, “Progress (in science) depends on the interplay of techniques, discoveries, and ideas, probably in that order of importance [21].

Some of the major research thrusts and needs include:

- Effects of sea level rise, salt water intrusion, and flooding on cycling of inorganic and organic contaminants such as metal (loid)s and nutrients
- Fate and transport of antibiotics, hormones, PFAS and other emerging contaminants
- Effects of warming of permafrost soils on carbon complexation with and release from soil minerals and emission of greenhouse gases
- Modeling that integrates spatial and temporal scales
- Advances in field-based spectroscopic techniques
- Development and deployment of real-time sensors
- Real-time investigations of soil chemical reactivity at the molecular scale
- Coupled physical, chemical, and biological process studies
- Mechanisms of mineral/microbe interactions
- Advances in understanding light element chemistry, e.g., Al, B, Ca, and S in soils using new tender and soft X-ray techniques

## Challenges and opportunities in environmental soil chemistry research

While there are so many exciting opportunities in the next decade in environmental soil chemistry research, there are still outstanding challenges now and in the future. One of the hallmarks of some of the most pioneering research in the field has been fundamental basic research. Soil chemists in the past were able to focus on a few areas for multiple periods such that they could “dig deeply” into the topic and become leading experts. This was made possible due to a continuity in funding for multiple periods. Over the past decade or more, institutional funding has decreased along with funding from federal agencies and the private sector. Additionally, the focus areas of research that funders support also change frequently which causes scientists to shift on a frequent basis from one topic to another. Thus, it is difficult to work in a particular area for an extended period of time and be viewed as an expert. Such shifting in focus could deleteriously impact the long-term reputation of a scientist. My thoughts on the critical need for basic, fundamental research and taking a deep dive into a particular area are best summed up by Albert Einstein, who stated, “I have little patience with scientists who take a board of wood, look for its thinnest part, and drill a great number of holes where drilling is easy”. There has also been a tendency for funding agencies to create large team science programs where multiple investigators, often from different institutions, pursue research on an interdisciplinary project. There is no question that many of the big challenges and opportunities in environmental soil chemistry research require an interdisciplinary approach. While soil chemists must focus on a few areas in depth at the fundamental level, they should take advantage of the exciting research opportunities that cross academic disciplines. However, the downside for individual scientists who pursue primarily large interdisciplinary science projects, especially early career scientists, is that their individual research products, i.e., refereed papers, often are not given the degree of credit that would result from publications that included only them and their students/postdocs. The overall significant research impacts from large team science recently was questioned. In a recent paper by Wu et al. [22], more than 64 million papers, patents and software products over a period of 1954–2014 were examined. The results showed that small teams of scientists tended to produce impactful results and ideas while large teams developed existing ideas.

The environmental challenges we face are daunting. However, with challenges there are opportunities. The advances in analytical tools and cyberinfrastructure offer exciting opportunities for soil chemists to tackle and help solve some of the most pressing issues facing humankind. In short, the future of environmental soil chemistry is indeed bright.
